# External Ophthalmomyiasis Caused by *Lucilia sericata* (Diptera: Challiphoride) Larva

**Published:** 2017

**Authors:** Roghayeh NOROUZI, Arman MANOCHEHRI, Saman ZARRIN

**Affiliations:** 1. Dept. of Pathobiology, Faculty of Veterinary Medicine, University of Tabriz, Tabriz, Iran; 2. Medical Laboratory, Shahid Ghazi Hospital, Kurdistan, Sanandaj, Iran

**Keywords:** Ophthalmomyiasis, *Lucilia sericata*, Iran

## Abstract

Myiasis is an animal or human pathogenic condition initiated by parasitic dipterous fly larvae feeding in the host’s necrotic or living tissue. Here we report a case of external ophthalmomyiasis caused by *Lucilia sericata* in a 78-yr-old with a vascular tumor of the retina and surgery history, from Bijar City of Kurdistan Province, Iran in 2015. Associated symptoms included right eye pain with mucoid ocular discharge, headache, sensing the presence of a foreign body in the eye and itching. Examination revealed a *L. sericata* Larva in his right eye. Infestation of ocular tissue by fly larvae (ophthalmomyiasis) progresses after retinal surgery and can destroy orbital tissues within days, especially in patient with poor hygienic conditions. Treatment consists of removal of the larvae and surgical debridement. Following removal of larva, the symptoms completely resolved within a few hours and remained asymptomatic several weeks later.

## Introduction

Myiasis is the infestation of tissues of animals or man by parasitic dipterous fly larvae. The most common site of infestation is the skin wound. “Less common sites are eyes, nose, paranasal sinuses, throat, and urogenital tract” (1).

Myiasis is most likely to occur in areas with overcrowding and hygienic conditions that are substandard but can also occur in hospitals and nursing homes (2).

Ophthalmic myiasis or ophthalmomyiasis is classified as either external or internal. “Ophthalmo myiasis externa is the result of infestation of the conjunctiva by the larval form or maggots of flies from the order Diptera” (3, 4).

Ophthalmomyiasis has a worldwide distribution because numerous genera and species have been implicated in intraocular disease. This fly larva may be seen in the anterior segment, vitreous, and sub retinal space. Ophthalmomyiasis has been reported from Iran (4, 5) and other countries of the world especially in developing countries (6–11). Most of these reports are limited to one case. *L. sericata*, also known as the green bottle fly, is a fly in the Calliphoridae family. The larvae of this species can cause myiasis, as well as accidental myiasis (12).

We report a case of external ophthalmomyiasis in an old man in Bijar City of Kurdistan Province, Iran referred for evaluation of a sub retinal lesion in the right eye. The patient had a history of vascular tumor of the retina and he operated but hygienic conditions that are poor.

## Case Report

A 78-yr-old man, from Bijar City, Iran, with a vascular tumor of the retina and surgery history, presented to the Cornea Associates of Imam Hossein Hospital, Bijar on May 2015 reporting that worm came out of his eye.

Informed consent was taken from the patient and the study was approved by the university.

Related symptoms included continuous bilateral eye pain with mucoid ocular discharge and headache over the five-day period before presentation. The first symptom, sensing the presence of a foreign body in the eye and itching, had always appeared abruptly. The eye involvement was unilateral and extra ocular with motile larvae present in the bulbar conjunctiva.

There was history of previous ocular surgeries, in his right eye was seen at the Imam Hossein Hospital, Bijar. The conjunctiva was markedly injected, and there was a moderate more of unclear discharge. Examination of the left eye was negative but right eye with the slidamp revealed white mobile wormlike object. As soon as the beam of the slit lamp was focused on them, they moved away. After instillation of a local anesthetic, irrigation was attempted with normal saline. The specimen was examined at the Imam Hossein Hospital District Department of Parasitology.

The maggot was removed from the right, placed in 10% neutral-buffered formalin, and sent to a laboratory for further recognition. A cylindrical vermiform maggot measuring 13mm in length and 3mm in diameter was observed under the dissecting microscope. The specimen was immediately washed in phosphate-buffered saline, pH 7.4, and cleared in graded solutions of glycerol (up to 80%). The larva was identified as third instar of *L. sericata* (Diptera: Calliphoridae).

**Fig. 1:**
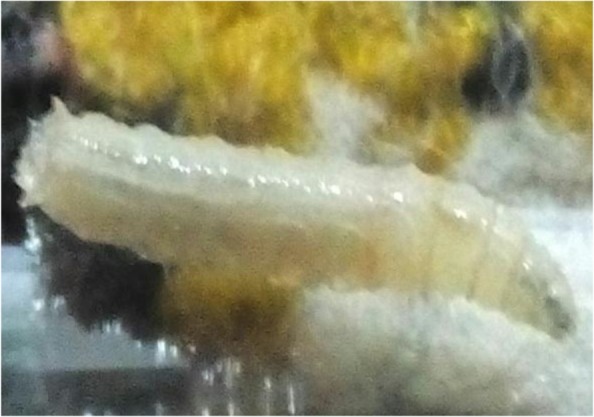
A third-stage larva of the *L. sericata*

**Fig. 2:**
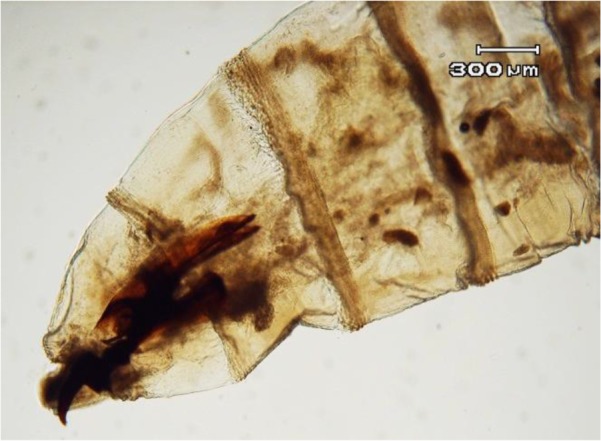
Anterior end of larva, 40X. Note the terminaloral hooks (Cephaloskeleton)

**Fig. 3:**
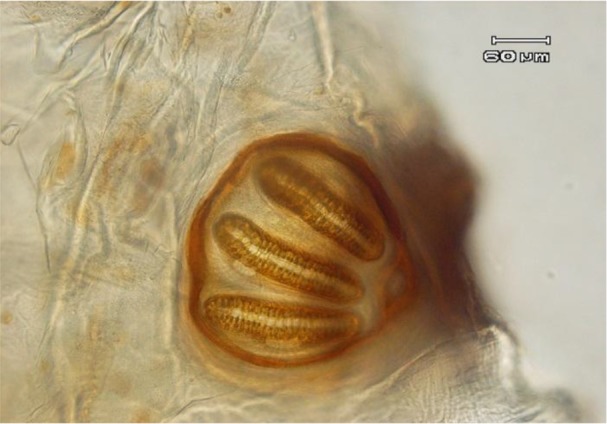
Posterior spiracle of larva, 40X

**Fig. 4:**
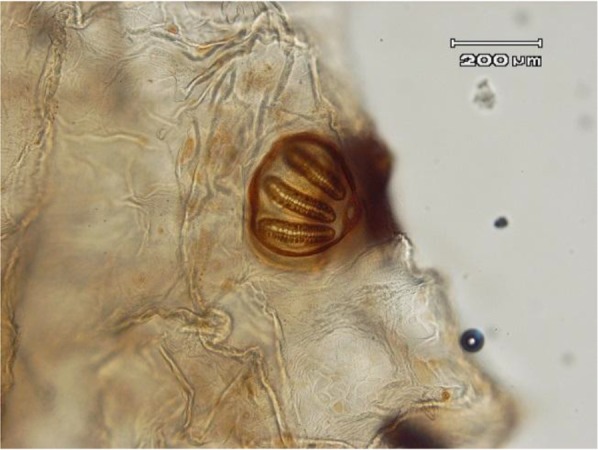
Posterior spiracle of larva, 10X

The body of larvae in necrophagous Calliphoridae family follows the common pattern for Calyptrata, three thoracic segments, seven abdominal segments, and the anal division (Fig. 1). The larvae have a pair of strong mandibles for eating food substrates and cephaloskeleton without sclerotized area below the posterior tip of ventral cornua (Fig. 2) (13). Third instar of *L. sericata* is easily recognizable from the other instars by the presence of anterior spiracles (Fig. 3 and 4). Mobile camera (Sony Xperia Z1) was used to take picture from immediately extracted larvae, but the rest of photos were taken with the Olympus EX60 microscope camera in parasitology laboratory.

Following removal of larva, the symptoms completely resolved within a few hours and remained asymptomatic several weeks later.

## Discussion

There are over 85000 species in Diptera order, but only a few genera and species have caused ophthalmomyiasis. In most reported cases of ophthalmomyiasis, classification of larvae performed by their posterior spiracle and cephaloskeleton structure (14).

The most cases of ophthalmomyiasis external are caused by *Dermatobiahominis*, endemic to tropical countries, *Oestrus ovis* (sheep bot fly), latrine fly (*Fannia*), house fly (*Muscadomestica*), and cattle botfly (*Hypoderma*) (11,12). Human ophthalmomyiasis must be expected in areas where maggots are prevalent, i.e. in areas where sheep, cattle, horses, and deer are to be found. Human beings are affected mainly in those areas where the density of sheep is relatively low compared with that of human beings (11) and our patient lived in rural areas and in close contact with small ruminants.

Symptoms, such as severe eye pain, redness, foreign body sensation, irritation, lacrimation, lids swelling, and predominance of male patients presented in this study are similar to those described in other reports (4, 8, 15). Rhinorrhea, reported in a few studies (16, 17), may point out to allergic reaction in addition to local irritation induced by fly larvae. Generally, these flies do not parasitize the hosts, they just lay their eggs in necrotic tissues, corpses and open wounds especially in animals, therefore, humans are the accidental hosts and trauma is known as an important risk factor (18). “*L. sericata* prefers humid and warm weather and it found in the tropical regions, therefore, it can be found around coast-land dry regions” (19).

More scattered cases have been reported since then from Italy, Russia, Serbia, India, Africa, America, and Oman (8, 9). Although only a few ophthalmomyiasis cases have been reported from Iran (5, 6) but this is the first ophthalmomyiasis due to *L. sericata* from Kurdistan Province in Iran.

The treatment of all types of ophthalmomyiasis is directed on removing the larvae and surgical debridement to prevent secondary infections (20–22).

## Conclusion

Myiasis should be considered as an occupational disease among farmers and shepherds. Awareness of larval conjunctivitis in rural areas, especially during spring and summer, leads to the more prompt diagnosis, and institution of specific therapy for the disease.
